# Screening of biomarkers of drug resistance or virulence in ESCAPE pathogens by MALDI-TOF mass spectrometry

**DOI:** 10.1038/s41598-019-55430-1

**Published:** 2019-12-12

**Authors:** Samantha Flores-Treviño, Elvira Garza-González, Soraya Mendoza-Olazarán, Rayo Morfín-Otero, Adrián Camacho-Ortiz, Eduardo Rodríguez-Noriega, Adrián Martínez-Meléndez, Paola Bocanegra-Ibarias

**Affiliations:** 1 Universidad Autónoma de Nuevo León, Hospital Universitario Dr. José Eleuterio González, Monterrey, Nuevo León, Mexico; 2 Instituto de Patología Infecciosa y Experimental, Centro Universitario de Ciencias de la Salud, Hospital Civil de Guadalajara Fray Antonio Alcalde, Universidad de Guadalajara, Jalisco, Mexico

**Keywords:** Mass spectrometry, Clinical microbiology, Infectious-disease diagnostics, Pathogens

## Abstract

Rapid identification and characterisation of drug-resistant bacterial pathogens have an important role in diagnostic and antimicrobial stewardship. Response time in the diagnosis of not only the etiological agent but also in antimicrobial susceptibility results is of utmost importance in patient treatment. In this study, matrix-assisted laser desorption ionisation–time of flight (MALDI-TOF) mass spectrometry (MS) was used to screen for biomarkers of ESCAPE (vancomycin-resistant *Enterococcus faecium*, methicillin-resistant *Staphylococcus aureus*, hypervirulent NAP1/ribotype 027 *Clostridioides* [*Clostridium*] *difficile*, multidrug resistant *Acinetobacter baumannii*, multidrug resistant *Pseudomonas aeruginosa*, and carbapenem-resistant *Enterobacteriaceae*) pathogens to predict antimicrobial resistance or hypervirulence. Several biomarkers of drug-resistant genotypes in *S. aureus, A. baumannii, P. aeruginosa*, and *K. pneumoniae*, as well as hypervirulence in *C. difficile*, were detected. The fastest possible susceptibility testing with MALDI-TOF MS is simultaneous detection of a characteristic drug-resistant peak and species identification in the same spectra generated in routine processing. According to our approach, resistance or virulence biomarker peaks can be identified while performing routine microbiology analysis, and no additional assays nor prolonged incubation time is needed. Outstanding biomarker peaks detected in our study should be further analysed by additional methods to identify the specific proteins involved.

## Introduction

The emergence of drug-resistant bacterial strains has been accelerated by both antibiotic overuse and reduced infection control. In 2013, the Centers for Disease Control and Prevention issued a report with the most concerning drug-resistant threats in the United States^1^. Of those mentioned, the most notable are pathogens predominately associated with healthcare-associated infections (HAI)^2^.

Reports by the Infectious Diseases Society of America have highlighted the importance of specific nosocomial drug-resistant pathogens^3^. Designated by the acronym ESKAPE, this group comprises the following pathogens: vancomycin-resistant *Enterococcus faecium* (VREfm), methicillin-resistant *Staphylococcus aureus* (MRSA), carbapenem-resistant *Klebsiella pneumoniae*, multidrug resistant (MDR) *Acinetobacter baumannii*, MDR *Pseudomonas aeruginosa*, and carbapenem-resistant *Enterobacter cloacae*^3,4^. These pathogens are of considerable concern due to their high frequency in HAIs^5,6^.

*Clostridioides* [*Clostridium*] *difficile* infection is one of the most common HAIs in hospitalised patients receiving antimicrobial therapy. In particular, the infection of hypervirulent *C. difficile* NAP1/ribotype 027 is associated with severe disease and high mortality rates^7,8^. In recent years, hospitals have reported a rise in *C. difficile* infection rates^9^. Thus, researchers have suggested amending the ESKAPE group by replacing *K. pneumoniae* with *C. difficile* and adding the entire *Enterobacteriaceae* family (instead of only *K. pneumoniae* and *E. cloacae*), changing the acronym to ESCAPE^10,11^.

Rapid identification and characterisation of drug-resistant bacterial pathogens have an important role in diagnostic and antimicrobial stewardship. The mission of the Antibacterial Resistance Leadership Group is to reduce the public health threat of antibacterial resistance^12^, which can be achieved by rapid detection of MDR isolates in clinical diagnostic laboratories. Matrix-assisted laser desorption ionisation–time of flight (MALDI-TOF) mass spectrometry (MS) is a fast and reliable technique that has been implemented in clinical microbiology laboratories to replace or complement conventional phenotypic identification for most isolated bacterial strains^13^. Although the report of antibiotic susceptibilities in the laboratory is necessary, the rapid determination of antimicrobial resistance through the use of MALDI-TOF MS is not yet available^14^. Response time in the diagnosis of not only the etiological agent but also in antimicrobial susceptibility results is of utmost importance in patient treatment. The fastest possible susceptibility testing with MALDI-TOF MS is simultaneous detection of a characteristic drug-resistant peak and species identification in the same spectra generated in routine processing^15^. Therefore, this study aimed to screen for biomarkers using MALDI-TOF MS in ESCAPE pathogens to predict antimicrobial resistance or hypervirulence.

## Results

### Selected genes and virulence factors

Among the *E. faecium* isolates, 53.1% (34/64) were vancomycin-resistant, and 46.9% (30/64) were vancomycin-susceptible (Table 1). The *vanA* gene was detected in all (34/34) VREfm isolates, whereas the *vanB* gene was not detected in any of the strains.

**Table 1:** Distribution of potential peaks detected, protein/peptide assignment and diagnostic utility.

Species, virulence/resistance phenotype (n)	Theoretical peaks (p ≤ 0.05)	Potential biomarkers				Protein	Mascot score	Protein sequence coverage (%)	Sensitivity (%)	Specificity (%)	PPV (%)	NPV (%)
	*n*	*N*	*m/z*	AUC	*p*							
*E. faecium*, vancomycin-resistant (34) *vs* vancomycin-susceptible (30)	70	0	NA	NA	NA	NA	NA	NA	NA	NA	NA	NA
MRSA (36) *vs* MSSA (31)	45	1	4,594 (P)	0.90	<0.001	50 S ribosomal protein L28	26	64	83.3	96.8	96.8	83.3
*C. difficile*, 027 ribotype (57) *vs* non-027 (36)	76	2	6,654 (A)	0.96	<0.001	30 S ribosomal protein S20	31	67	100.0	91.7	95.0	100.0
			6,712 (P)	0.98	<0.001	30S ribosomal protein S21	34	98	100.0	91.7	95.0	100.0
***A. baumannii***, ***bla***_**OXA-24**_ **or** ***bla***_**OXA -58**_ **(33)** ***vs***												
non-*bla*_OXA-24_ or *bla*_OXA -58_ (35)	2	0	NA	NA	NA	NA	NA	NA	NA	NA	NA	NA
*A. baumannii, bla*_OXA-24_ (26) *vs bla*_OXA -58_ (7)	6	2	6,304 (P)	0.99	<0.001	NADH-quinone oxidoreductase subunit K	29	56	85.7	100.0	100.0	96.3
			6,332 (A)	0.99	<0.001	NADH-quinone oxidoreductase subunit K	29	56	100.0	100.0	100.0	100.0
*P. aeruginosa*, MDR (27) *vs* non-MDR (28)	61	3	2,726 (P)	0.81	<0.001	UPF0270 protein Pfl01_4103	20	32	75.0	66.6	70.0	72.0
			5,455 (P)	0.81	<0.001	UPF0391 membrane protein Patl_1732	32	98	82.1	51.9	67.7	66.7
			5,742 (P)	0.84	<0.001	Not determined	NA	NA	85.7	40.7	60.0	73.0
			2,726, 5,455, and 5,742 (P)			NA	NA	NA	75.0	74.1	75.0	74.1
*K. pneumoniae*, carbapenem-resistant (49) *vs* carbapenem-susceptible (20)	96	0	NA	NA	NA	NA	NA	NA	NA	NA	NA	NA
*K. pneumoniae*, carbapenem- and colistin-resistant (16) *vs* carbapenem- and colistin-susceptible (20)	75	1	6,100 (A)	0.86	<0.001	Not determined	NA	NA	93.8	55.0	62.5	91.7

A: absence; AUC: area under the curve; MDR: multidrug resistant; MRSA: methicillin-resistant *S. aureus*; MSSA: methicillin-susceptible *S. aureus*; NPV: negative predictive value; P: presence, PPV: positive predictive value.

Of the *S. aureus* isolates, 53.7% (36/67) were MRSA (cefoxitin-resistant) and *mecA* positive; 46.3% (31/67) were methicillin-susceptible *S. aureus* (MSSA) and *mecA* negative (Table 1).

For *C. difficile* isolates, 61.3% (57/93) were ribotypes 027 and 38.7% (36/93) were non-027 ribotypes (Table 1). The following non-027 ribotypes were identified: 001 (*n* = 17; 47.2%), 106 (*n* = 4; 11.1%), 003 *(n* = 3; 8.3%), 176 (*n* = 3; 8.3%), 002 (*n* = 1; 2.8%), 012 (*n* = 1; 2.8%), 014 (*n* = 1; 2.8%), 017 (*n* = 1; 2.8%), 019 (*n* = 1; 2.8%), 020 (*n* = 1; 2.8%), 076 (*n* = 1; 2.8%), 220 (*n* = 1; 2.8%), and 353 (*n* = 1; 2.8%).

For *A. baumannii* isolates, 45.6% (31/68) were carbapenem resistant. Class D *bla*_OXA_ carbapenemase genes *bla*_OXA-24_ and *bla*_OXA-58_ were detected in 26/68 and 7/68 isolates, respectively. In addition, *bla*_OXA-58_ was detected in two carbapenem-susceptible isolates. As expected, the *bla*_OXA-51_ gene was detected in all (68/68) strains. Neither class D *bla*_OXA-23_, nor encoding metallo-β-lactamases genes *bla*_NDM_, *bla*_VIM_, and *bla*_IMP_ genes were detected.

Among *P. aeruginosa* isolates, 49.1% (27/55) were MDR, and 50.9% (28/55) were non-MDR. The *bla*_VIM_ and *bla*_IMP_ genes were not detected.

For *K. pneumoniae* isolates, 72.6% (49/69) were carbapenem resistant, and 27.4% (20/69) were carbapenem susceptible. Of the carbapenem-resistant isolates, 16/49 (32.7%) were colistin-resistant; none of the carbapenem-susceptible isolates presented colistin resistance. The *bla*_NDM_ gene was detected in all the carbapenem-resistant isolates. Neither *bla*_KPC_, *bla*_IMP_, *bla*_VIM_, nor *bla*_OXA-48_ were detected.

### Potential biomarkers

The generated protein profile of each isolate spot had mass-charge ratio (*m/z*) values between 2,000 and 20,000. A MS spectrum sample of each species is shown in Supplementary Fig. S1. Classification models were generated based on the GA, SNN, and QC algorithms, in which at least one of them yielded adequate cross-validation and recognition capability values for vancomycin-resistant vs vancomycin-susceptible *E. faecium* (90% and 98%, respectively), *C. difficile* ribotype 027 vs non-027 (90% and 96%, respectively), *mecA* positive vs *mecA* negative (87% and 98%, respectively), carbapenem-resistant vs carbapenem-susceptible *K. pneumoniae* (78% and 90%, respectively), MDR vs non-MDR *P. aeruginosa* (76% and 89%, respectively), carbapenem/colistin-resistant vs carbapenem/colistin-susceptible *K. pneumoniae* (67% and 83%, respectively), *bla*_OXA-24_ vs *bla*_OXA-58_ carbapenem-resistant *A. baumannii* (59% and 51%, respectively), and *bla*_OXA-24/58_ positive vs *bla*_OXA-24/58_ negative *A. baumannii* (50% and 72%, respectively). A complete description of all classification analyses is shown in Supplementary Table S2. The ability of each algorithm to classify spectra from the same set of samples was used to perform an external validation. At least one of the models was able to allocate most of the spectra of the validation set to their corresponding phenotype/genotype profile (Table S2).

In the initial screening and after group comparisons (phenotype/genotype absent or present), statistically significant peaks (*p ≤ *0.05) were detected for carbapenem-resistant *K. pneumoniae* (n = 96), *C. difficile* ribotype 027 (n = 76), carbapenem and colistin-resistant *K. pneumoniae* (n = 75), VREfm (n = 70), MDR *P. aeruginosa* (n = 61), MRSA (n = 45), *bla*_OXA-58_ *A. baumannii* (n = 6), and carbapenem-resistant *A. baumannii* (*bla*_OXA-24_ or *bla*_OXA-58_; n = 2) (Table 1). The complete list describing all significant peaks is shown in Supplementary Dataset S3.

The distribution of each strain after PCA based analysis per phenotype/genotype of all species is shown in Supplementary Fig. S4. The distinguishing capability between drug-resistance (or virulence) phenotype/genotype profiles in the isolates based on their peptide mass fingerprints was highly variable.

These statistically significant peaks were selected for further analysis which were the analysis of the peak area/intensity of the spectra, the coefficient of variation and the value of area under the curve [AUC] of each peak. When all three criteria were met, these peaks were considered as “potential biomarkers peaks”.

When analysis VREfm, no potential biomarkers were finally selected.

**Figure 1:**
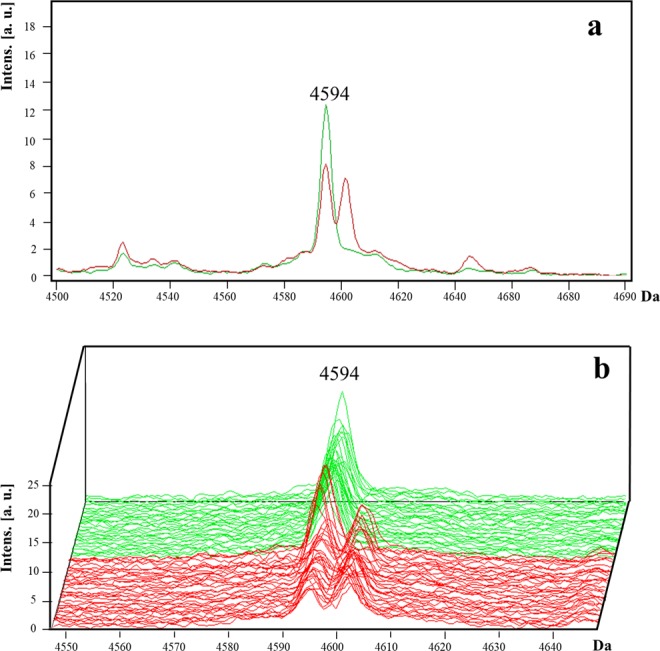
Representative mass spectra of peak *m/z* 4,594 in *S. aureus*. The peak *m/z* 4,594 is presented as a doublet in MRSA isolates (red line) and as a singlet in MSSA isolates (green line). The average spectrum of each group of peak *m/z* 4,594 (**a**) and the spectra of all the analysed isolates (**b**) are shown.

For *S. aureus* isolates, one peak with a *m/z* of 4,594 (with a value of area under the receiver operating characteristics curve [AUC-ROC] of 0.82; *p < *0.001) was selected (Table 1). This peak was present as a singlet in the MSSA isolates; however, in the MRSA isolates, it displayed a splitting pattern as a doublet (Fig. 1). The presence of the peak *m/z* 4,594 as a doublet had a sensitivity of 83.3% and a specificity of 96.8% for the detection of methicillin-resistant phenotype (Table 1). Furthermore, a peak previously reported as a potential biomarker (*m/z* 2,415) was detected in 63.9% (23/36) of the MRSA isolates^16^.

**Figure 2:**
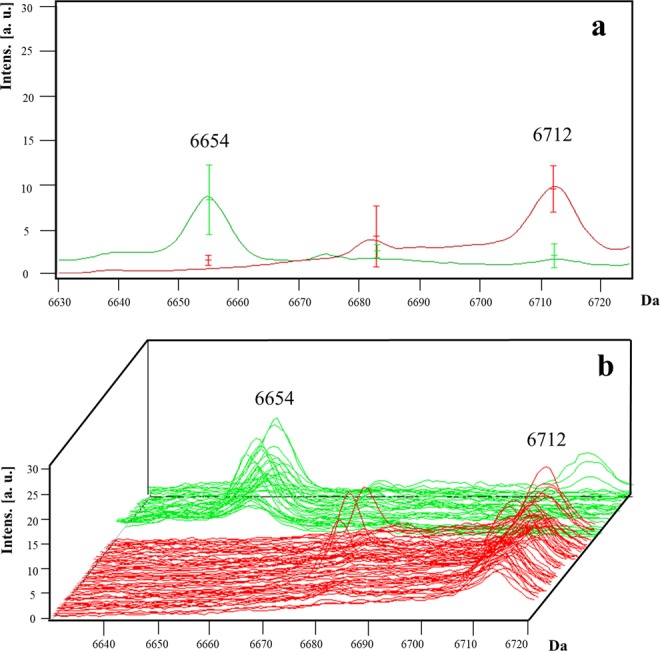
Representative mass spectra of peaks *m/z* 6,654 and 6,712 in *C. difficile*. The peak *m/z* 6,654 is present in non-027 ribotype isolates (green line) and is absent in 027 and 176 ribotype (red line). By contrast, the peak *m/z* 6,712 is present in 027 and ribotypes but absent in non-027 ribotypes. The average spectrum of each group of peaks *m/z* 6,654 and 6,712 (**a**) and the spectra of all the analysed isolates (**b**) are shown.

For *C. difficile* isolates, peaks were selected by strain: the peak at *m/z* 6,654 (AUC = 0.96) was present only in non-027 strains, except for the 176 ribotype; and the peak at *m/z* 6,712 (AUC = 0.99; *p < *0.001) was present only in 027 and 176 strains (Fig. 2). To detect the hypervirulent 027 ribotype, the absence of peak *m/*z 6,654 and presence of peak *m/z* 6,712 presented a sensitivity of 100%, specificity of 91.7%, and PPV of 95%.

**Figure 3:**
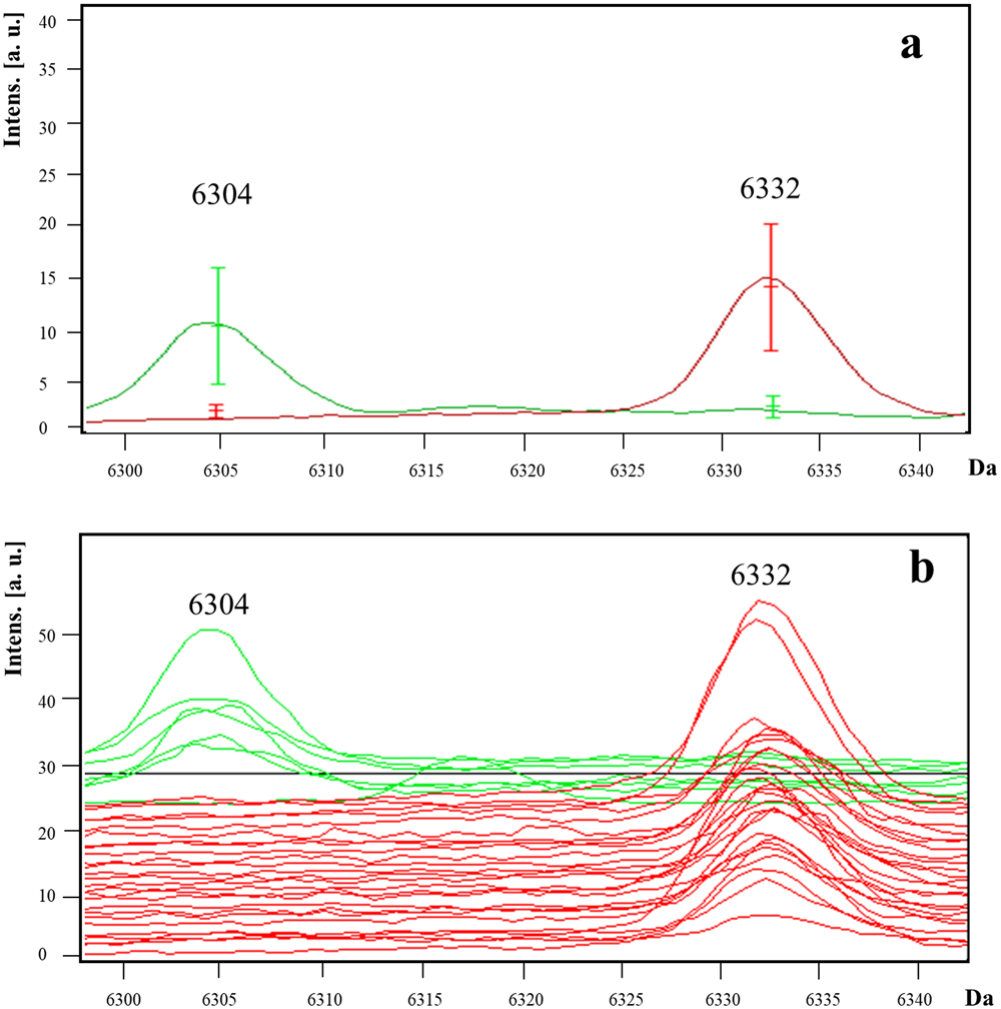
Representative mass spectra of peaks *m/z* 6,304 and 6,332 in *A. baumannii*. The peak *m/z* 6,304 is present in *bla*_OXA-58_ isolates (green line) and is absent in *bla*_OXA-24_ isolates (red line). By contrast, the peak *m/z* 6,332 is present in *bla*_OXA-24_ but absent in *bla*_OXA-58_ isolates. The average spectrum of each group of peaks *m/z* 6,304 and 6,332 (**a**) and the spectra of all the analysed isolates (**b**) are shown.

*A. baumannii* bla_OXA-24_ were compared with bla_OXA-58_ isolates, and two peaks were selected: the peak at *m/z* 6,304 (AUC = 0.99; *p < *0.001) was present only in *bla*_OXA-58_-positive isolates (specificity, and positive predictive value [PPV] of 100%), and the peak at *m/z* 6,332 (AUC = 0.99, *p < *0.001) was present only in *bla*_OXA-24_-positive isolates (sensitivity, specificity, PPV, and negative predictive value [NPV] of 100%) (Table 1) (Fig. 3). The comparison of *bla*_OXA-58_-positive with *bla*_OXA-58_- plus *bla*_oxa-24_-negative isolates yielded a statistically significant peak at *m/z* 6,332 (*p = *0.016). The comparison of isolates that harboured either *bla*_OXA-24_ or *bla*_OXA-58_ with isolates that harboured exclusively *bla*_OXA-51_ genes showed no potential biomarkers.

**Figure 4:**
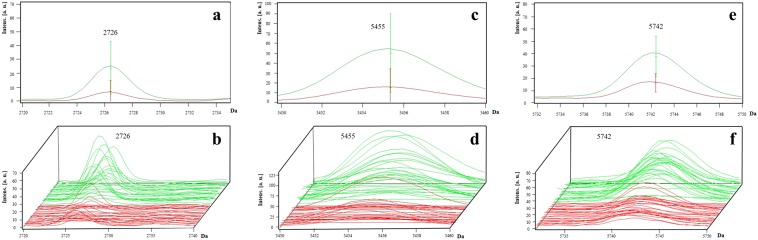
Representative mass spectra of peaks *m/z* 2,726, 5,455 and 5,742 in *P. aeruginosa*. Three peaks *m/z* 2,726, 5,455, and 5,742 were present in the majority of non-MDR isolates (green line) and at higher intensities than those in MDR isolates (red line). The average spectrum of each group of peaks *m/z* 2,726 (**a**), 5,455 (**c**), and 5,742 (**e**) and the spectra of all the analysed isolates for peaks *m/z* 2,726 (**b**), 5,455 (**d**), and 5,742 (**f**) are shown.

For *P. aeruginosa* isolates, three potential biomarkers were detected at peaks *m/z* 2,726 (AUC = 0.81; *p < *0.001), 5,455 (AUC = 0.81; *p < *0.001), and 5,742 (AUC = 0.84; *p < *0.001), all of which predominated in the non-MDR isolates (Fig. 4). The presence of all three peaks increased the specificity to 74.1%, PPV to 75%, and NPV to 74.1% for the detection of non-MDR isolates (Table 1).

**Figure 5:**
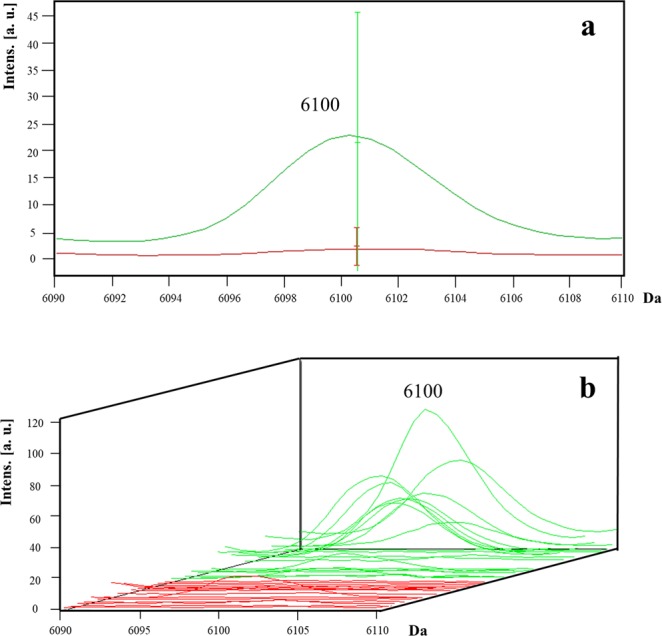
Representative mass spectra of peaks *m/z* 6,100 in *K. pneumoniae*. The peak *m/z* 6,100 is absent in the carbapenem- and colistin-resistant isolates (red line) and is present in the majority of the carbapenem- and colistin-susceptible isolates (green line). The average spectrum of each group of peak *m/z* 6,100 (**a**) and the spectra of all the analysed isolates (**b**) are shown.

No potential biomarkers were detected for the carbapenem-resistant *K. pneumoniae* isolates. However, the absence of peak *m/z* 6100 (AUC = 0.88) had a sensitivity of 93.8% and a NPV of 91.7% for the detection of carbapenem and colistin-resistant *K. pneumoniae* (Fig. 5) (Table 1).

### Peptide/protein identity assignment

Assignment of possible peptide/protein identity of all the identified potential marker peaks is shown in Table 1. The *m/z* 4,594 peak of *S. aureus* was assigned to the 50 S ribosomal protein L28 with MASCOT score of 26 and protein sequence coverage of 64% Both the *m/z* 6,654 and 6,712 peaks of *C. difficile* were assigned to the 30 S ribosomal protein (S20 and S21, respectively), with 31–34 score and 67–98% protein sequence coverage. Both the *m/z* 6,304 and the *m/z* 6,332 peak of *A. baumannii* was assigned to a NADH-quinone oxidoreductase subunit K with a score of 29 and 56% of protein sequence coverage for each peak. In *P. aeruginosa*, the *m/z* 2,726 peak was assigned to a UPF0270 protein Pfl01_4103 with a score of 20 and 32% of protein sequence coverage, and the *m/z* 5,455 peak was assigned to a UPF0391 membrane protein Patl_1732 with a score of 32 and 98% of protein sequence coverage. The *m/z* 5,742 peak of *P. aeruginosa* and the *m/z* 6,100 peak of *K. pneumoniae* could not be determined.

## Discussion

MALDI-TOF MS is now widely used in clinical microbiology for bacterial identification, taxonomy, and strain typing^17^. One application in development is the detection of antibiotic resistance in bacteria. Thus far, several approaches have been proposed in MALDI-TOF MS to detect antimicrobial resistance such as the detection of the entire cell profile, enzymatic activity by antibiotic hydrolysis, or resistance proteins within the cell^18–21^. In this study, we identified several potential biomarkers of drug-resistant genotypes in *S. aureus, A. baumannii, P. aeruginosa*, and *K. pneumoniae*, as well as hypervirulence in *C. difficile*, using a direct approach.

Regarding *C. difficile*, 2 out of 73 peaks reached statistical significance, wherein isolates were divided into 027 and non-027 ribotype groups. The peak *m/z* 6,654 was absent in 027 and 176 ribotypes, whereas the peak *m/z* 6,712 was present in both ribotypes. These findings complicate the differentiation of hypervirulent strains (027) from non-hypervirulent strains (176). However, ribotype 176 shares many similarities with 027, such as the presence of binary toxin genes and a single-base-pair deletion at nucleotide 117 in the gene that encodes a negative regulator of toxin production^22^, in such way that is actually difficult to differentiate both ribotypes in most methodologies^23,24^. Nevertheless, severe disease outcomes and mortality are associated with ribotype 176^25^ and in clinical epidemiology, hypervirulent strains should be notified^24^. In our work, 027 strains were differentiated from other non-027 strains, such as 001, 002, 003, 012, 014, 017, 019, 020, 076, 106, 220, and 353 ribotypes. It is highly likely (98% of protein sequence coverage) that 30S ribosomal protein was assigned to both peaks, which binds directly to 16S ribosomal RNA. In addition, both peaks had excellent AUC values (0.96–0.99), sensitivity, specificity, PPV, and NPV, all of which indicate a good performance of a biomarker.

MRSA is one of the most studied pathogens of the ESCAPE group regarding MALDI-TOF analysis^15,19,21,26–28^. A number of studies have used MALDI-TOF MS to discriminate clonal complexes of MRSA (CC5, CC8, CC22, CC30, and CC45)^29^. In the present study, the peak *m/z* 4,594 had a high specificity of 96.8% to detect MRSA isolates. The 50S ribosomal protein was most probably assigned to this peak. Different protein synthesis inhibitors, such as macrolides, ketolides, lincosamides, and B-type streptogramins, prevent the 50S particle from being formed in growing cells^30^. To our knowledge, this is the first report of a potential biomarker peak for MRSA isolates. In addition, a *m/z* 2,415 peak was detected in the MRSA isolates (not shown). This peak was previously reported in MRSA isolates with the class A *mec* gene complex^16^, though in our study the characterisation beyond the *mecA* gene detection was not performed.

Few reports have included the analysis of drug-resistant *A. baumannii* through MALDI-TOF MS, and although promising, their results were obtained using the carbapenem hydrolysis assay, not the direct approach^15^. In our study, two potential biomarker peaks were detected for MDR *A. baumannii*: *m/z* 6,304 and *m/z* 6,332. Both peaks had high AUC values (0.99) and were present in *bla*_OXA-58_- and *bla*_OXA-24_-positive isolates, respectively. A NADH-quinone oxidoreductase subunit was likely assigned to both peaks. Type II NADH-quinone oxidoreductases of Gram-negative bacteria can be inhibited by polymyxin B and colistin^31^. The performance of peak *m/z* 6,332 as a biomarker improved when comparing *bla*_OXA-58_-positive with *bla*_OXA-58_-negative and *bla*_OXA-24_ isolates. Our results indicate the discriminatory power of MALDI-TOF MS to differentiate *bla*_OXA-58_ from *bla*_OXA-24_ isolates, and this may be applied directly in clinical microbiology.

A signal loss of three peaks was detected for MDR *P. aeruginosa* isolates in comparison to non-MDR isolates. All three peaks had acceptable individual AUC values (0.81–0.84), and interestingly, the inclusion of all three peaks increased their potential as biomarker peaks for the detection of non-MDR isolates (sensitivity, 75%; specificity, 74.1%; PPV, 75%; and NPV, 74.1%). Two of the peaks were likely assigned to hypotheticals proteins, such as UPF0270 protein Pfl01_4103 (*m/z* 2,726 peak) and UPF0391 membrane protein Patl_1732 (*m/z* 5,455 peak). In case the identified peaks were proteins, the signal loss could be due to a mutation introducing a premature stop codon or a frame-shift in a gene. As it is, we cannot assume they are indeed associated to proteins and other explanations must be considered. Growth conditions and sample preparation may also contribute to signal loss. Therefore, loss of a signal does not alone give distinctive information about the strain genotype and cannot be used as a reliable biomarker peak^29^.

One potential biomarker for carbapenem and colistin resistance in *K. pneumoniae* was identified at peak *m/z* 6,100, which could be used to detect this phenotype, giving its NPV (91.7%). The use and the resistance to polymyxins have increased in carbapenemase-producing *Enterobacteriaceae*. A MALDI-TOF MS–based method that detects polymyxin resistance-related modifications to lipid A (i.e., phosphoethanolamine addition) was previously developed in *E. coli*. This methodology can be performed in 15 min and simultaneously discriminates between chromosome- and plasmid-encoded resistance^32,33^. This methodology has an advantage over our approach, both in specificity and processing time.

In addition, we did not assess the frequency of the colistin resistance-associated *mcr* gene in our *Enterobacteriaceae* isolates. Our study population contained the *bla*_NDM_ gene only in the carbapenem-resistant *K. pneumoniae* isolates. Because the *bla*_KPC_ *K. pneumoniae* genotype has been previously identified by MALDI-TOF^34^, future studies should analyse a higher number of isolates and a wider diversity of drug resistance–associated genes of *K. pneumoniae*.

Previous studies have compared VRE mass spectra among *vanA* or *vanB* positive and negative groups, specifically on *E. faecium* isolates^15,21^. A number of peaks were detected among *vanA-*positive isolates^35,36^ as well as *vanB-*positive isolates^37^. In our study, we detected 70 potential peaks with significant difference (p ≤ 0.05), but no final potential biomarkers were detected. Failure to identify lineage-specific biomarker peaks in Gram-positive pathogens such as *E. faecium* has been reported previously^38^.

Changes in the distribution of peaks among strains suggest a peak shift, possibly corresponding to a slight protein weight modification due to an amino acid substitution after gene mutations. This shift can be used as a trustable biomarker to identify the strain genotype (either related to virulence or drug resistance), which was the approach used in this study^29^. According to our approach, resistance or virulence biomarker peaks can be identified while performing routine microbiology analysis, and no additional assays nor prolonged incubation time is needed.

The primary limitation of this study was the lack of validation studies. Predictive biomarkers for MALDI-TOF should be first identified with a training set of data and afterward should be validated on an independent set of data. This validation set should include isolates with geographic, temporal, and clonal diversity to ensure the validity of the peak biomarkers^29^. In addition, outstanding potential biomarker peaks detected in our study should be further analysed by additional methods to identify which proteins/peptide each peak corresponds to.

In conclusion, we identified several potential biomarker peaks suggestive of drug resistance in *S. aureus, A. baumannii, P. aeruginosa*, and *K. pneumoniae*, as well as hypervirulence in *C. difficile* using MALDI-TOF MS.

## Methods

### Clinical isolates

Selected ESCAPE clinical isolates obtained between January 2007 and December 2017 from the Hospital Civil de Guadalajara “Fray Antonio Alcalde” (Jalisco, Mexico) were included. The VITEK 2 system (Biomérieux, Craponne, France) was used according to the manufacturer’s instructions, and either identification card for Gram positive or negative were used for identification. Bacteria were grown on plates containing Trypticase soy agar with 5% sheep blood and incubated overnight at 35 °C. A suspension of bacteria from pure cultures was performed in a 0.45% sodium chloride solution. This suspension was adjusted to a McFarland standard of 0.5 and along with the ID card, introduced into the system, which automatically inoculated the card and incubated at 35 °C until the end of the test.

*A. baumannii* species identification was confirmed by polymerase chain reaction (PCR). Primers and PCR conditions were used as described previously^39,40^. First, rapid DNA extraction was performed, in which three to five bacterial colonies were suspended in 100 µl of sterile distilled water and heated at 95 °C for 15 min. After centrifugation at 13,500 rpm for 5 min, the supernatant was collected. A pair of primers, forward (F): 5′-CATTATCACGGTAATTAGTG-3′ and reverse (R): 5′-AGAGCACTGTGCACTTAAG-3′, was used for the amplification of an internal fragment from the 16S-23S rRNA intergenic spacer region of *A. baumannii*. A second pair of primers, F5′-CCTGAATCTTCTGGTAAAAC-3′ and R5′-GTTTCTGGGCTGCCAAACATTAC-3′, were used for the amplification of a highly conserved region of the *recA* gene of *Acinetobacter* genus. The reaction mixture contained 1X PCR buffer, 2 mM MgCl_2_, 0.2 mM of each dNTP, 200 nM of each primer, 1 U of AmpliTaq polymerase and 2 µl of DNA. PCR was initiated by denaturation for 5 min at 94 °C, followed by 30 cycles of 30 s at 94 °C, 30 s at 55 °C and 30 s at 72 °C with final extension for 7 min at 72 °C and a holding step at 4 °C until analysis. Next, 5 µl of the PCR products were electrophoresed on a 2.0% agarose gel prepared with 0.5x TBE buffer for 1 h at 130 V. The gel was stained with 10 µM ethidium bromide, and the PCR product bands were visualized using a UV transilluminator. Expected PCR products for the 16S-23S rRNA intergenic spacer region and *recA* gene were 208 and 425 bp, respectively.

Selected clinical isolates included *E. faecium* (n = 64), *S. aureus* (n = 67), *C. difficile* (n = 93), *A. baumannii* (n = 68), *P. aeruginosa* (n = 55), and *K. pneumoniae* isolates (n = 69).

### Antimicrobial susceptibility and phenotypic tests

Antimicrobial susceptibility was determined using the broth microdilution method according to the 2019 Clinical and Laboratory Standards Institute^41^. The following strains were evaluated for resistance to the corresponding drugs: *E. faecium*, vancomycin; *S. aureus*, cefoxitin and oxacillin; *A. baumannii*, imipenem and meropenem; *P. aeruginosa*, gentamicin, cefepime, ciprofloxacin, imipenem, and meropenem; *K. pneumoniae*, ertapenem, imipenem, meropenem and colistin. Strains were classified as MDR as described elsewhere, i.e. resistant to three or more antimicrobial classes^42^.

### Genotyping

*E. faecium* isolates were screened as previously described for the presence of *vanA* and *vanB* genes after rapid DNA extraction. Primers F5′-CATGAATAGAATAAAAGTTGCAATA-3′ and R5′-CCCCTTTAACGCTAATACGATCAA-3′ were used for the amplification of *vanA* gene (1,030 bp); and F5′-GTGACAAACCGGAGGCGAGGA-3′ and R5′-CCOGCCATCCTCCTGCAAAAAA-3′, for the *vanB* gene (433 bp)^43^. PCR conditions were 10 min at 95 °C, followed by 30 cycles of 30 s at 94 °C, 30 s at 58 °C and 30 s at 72 °C with final extension for 10 min at 72 °C.

The *mecA* gene (147 bp) was screened in all *S. aureus* isolates, using primers F5′- GTGAAGATATACCAAGTGATT-3′ and R5′- ATGCGCTATAGATTGAAAGGAT-3′^44^. PCR conditions were 5 min at 94 °C, followed by 30 cycles of 1 min at 94 °C, 1 min at 50 °C and 2 min at 72 °C with final extension for 10 min at 72 °C.

*A. baumannii* isolates were screened for class D *bla*_OXA_ carbapenemases, including *bla*_OXA-23_ (F5′-GATCGGATTGGAGAACCAGA-3′ and R5′-ATTTCTGACCGCATTTCCAT-3′, 501 bp)_,_ *bla*_OXA-24_ (F5′-GGTTAGTTGGCCCCCTTAAA-3′ and R5′-AGTTGAGCGAAAAGGGGATT-3′, 246 bp)_,_ *bla*_OXA-51_ (F5′-TAATGCTTTGATCGGCCTTG-3′ and R5′-TGGATTGCACTTCATCTTGG-3′, 353 bp)_,_ and *bla*_OXA-58_ (F5′-AAGTATTGGGGCTTGTGCTG-3′ and R5′-CCCCTCTGCGCTCTACATAC-3′, 599 bp)^45^, and metallo-β-lactamases genes including *bla*_VIM_ (F5′-ATGGTGTTTGGTCGCATATC-′3 and R5′-TGGGCCATTCAGCCAGATC-′3, 510 bp)^46^, *bla*_IMP_ (F5′-GGAATAGAGTGGCTTAAYTCTC-′3 and R5′-CCAAACYACTASGTTATCT-′3, 188 bp)^47^, and *bla*_NDM_ (F5′-GGAAACTGGCGACCAACG-3′ and R5′-ATGCGGGCCGTATGAGTGA-3′, 678 bp)^48^. *P. aeruginosa* isolates were screened for *bla*_VIM_, and *bla*_IMP_ genes. *K. pneumoniae* isolates were screened for *bla*_KPC_ (F5′-GCAGCGGCAGCAGTTTGTTGATT-3′ and R5′-GTAGACGGCCAACACAATAGGTGC-3′, 184 bp)^49^, *bla*_VIM_, *bla*_IMP_, *bla*_NDM_, and *bla*_OXA-48_ (F5′-TTCGGCCACGGAGCAAATCAG-3′ and R5′-GATGTGGGCATATCCATATTCATCGCA-3′, 240 bp)^49^. Conditions for multiplex PCR (*bla*_OXA-23,_ *bla*_OXA-24,_ *bla*_OXA-51,_ and *bla*_OXA-58_) were 5 min at 94 °C, followed by 30 cycles of 30 s at 94 °C, 30 s at 55 °C and 30 s at 72 °C, and 6 min at 72 °C. For *bla*_NDM_ and *bla*_VIM,_ PCR conditions were 5 min at 94 °C, followed by 30 cycles of 30 s at 94 °C, 60 s at 60 °C and 45 s at 72 °C, and 5 min at 72 °C. For *bla*_IMP_, PCR conditions were 5 min at 95 °C, 30 cycles of 30 s at 95 °C, 40 s at 52 °C and 50 s at 72 °C, and 5 min at 72 °C. For multiplex PCR *bla*_OXA-48_ and *bla*_KPC_, conditions were 5 min at 94 °C, 30 cycles of 30 s at 94 °C, 30 s at 60 °C and 30 s at 72 °C, and 5 min at 72 °C.

Ribotyping of *C. difficile* isolates included the amplification of the 16S-23S rRNA intergenic spacer region using primers 5′-GTGCGGCTGGATCACCTCCT-3′ and 5′-CCCTGCACCCTTAATAACTTGACC-3′^50^. PCR conditions were 95 °C for 15 min, followed by 24 cycles at 95 °C for 1 min, 57 °C for 1 min and 72 °C for 1 min, and 72 °C for 30 min. Capillary electrophoresis was performed at the *C. difficile* Ribotyping Network Reference Laboratory at Leeds Teaching Hospitals Trust (Leeds, UK).

### Mass spectra analysis

All selected strains were prepared for MALDI-TOF MS (Microflex LT system, Bruker Daltonics, Bremen, Germany) according to the manufacturer’s recommendation. For the direct colony method, an overnight culture grown on blood agar at 37 °C under aerobic conditions of each bacterial strain or from a culture grown on *C. difficile* agar (Neogen Corporation, MI) in anaerobic conditions for up to 24 h, were included. One colony of each bacteria was applied using sterile wooden toothpicks on a 96-spot stainless steel target plate (Bruker Daltonics, Bremen, Germany). After drying, 1 μl 70% formic acid was added and air-dried prior to adding 1 μl of a matrix solution, which was composed of a saturated solution of α-cyano-4-hydroxycinnamic acid 10 mg/mL in 50% acetonitrile (Sigma-Aldrich, Toluca, Mexico) and 2.5% trifluoroacetic acid (Sigma-Aldrich), which enables measurement of peptides and proteins from 0.7 to 20 kDa. Finally, spots were allowed to dry completely, and the target plate was introduced into the equipment. Samples were analysed by MALDI Biotyper 3.0 software for spectra profile match in the database.

All the isolates were classified according to manufacturer’s recommended score identification criteria. A score of 2.0 to 3.0 indicated reliable species-level identification, a score of 1.7 to 1.9 indicated reliable genus identification but questionable species-level identification, and a score of <1.7 indicated an unreliable identification.

### Potential marker peaks

The MS spectrum of each spot was analysed by the MALDI Biotyper V.3.1.66 with the most updated spectra library, V.7.0 (7,311 spectra). High-quality spectra were captured using the flexControl V.3.4 software (Bruker Daltonics). These spectra were then imported into the ClinProTools V.3.0 software (Bruker Daltonics) for recognition of mass spectra patterns between groups and preprocessed using the default parameters. Spectra were smoothed with 10 cycles of the Savitzky/Golay algorithm for 10 cycles with a width of 2 *m/z*. Baseline subtraction was performed with the Top Hat algorithm. Peak picking was performed on the average spectrum from each group, with a signal to noise threshold of 5. Peak selection was performed using the P-value T-test/ANOVA sort mode.

Group selection was based on the presence or absence of drug-resistance genotypes, except for *C. difficile* in which hypervirulent ribotypes determination was considered.

### Statistical analyses

The selection of potential biomarker peaks of drug-resistant phenotypes was performed using statistical analyses within the algorithms included in the ClinProTools software. Classification models were generated using the genetic algorithm (GA), supervised neural network (SNN), and quick classifier (QC) algorithms. The parameters were maximum of 5 peaks, maximum of 50 generations, k-nearest neighbor classification with 3 neighbors for GA; 1–25 peaks automatically detected for SNN, and QC (automatic peak detection 1–25 peaks, p-value T-test/ANOVA). The recognition capability and cross validation values were calculated to demonstrate the reliability and accuracy of the model.

The Anderson-Darling test was used to determine the distribution of the population (≤0.05: not normally distributed, >0.05, normally distributed). According to the results, either the t-test (normally distributed data) or the Wilcoxon test (not normally distributed data) were used to confirm significant differences between two classes (on the presence or absence of drug-resistance or virulence phenotype/genotypes). As the *p*-value provides a measure of the strength of an association, the lower it is, the better the respective peak signal can be used to discriminate the groups. If the *p*-value for the t-test or the Wilcoxon test was ≤0.05, the peak was confirmed to be significantly different.

Peaks that obtained statistical significance were selected for further analysis, which included evaluating the peak area/intensity of the spectra and the coefficient of variation of each peak; this analysis allowed the selection of potential peaks. ClinProTools also calculates a Receiver Operating Characteristic (ROC) curve for each peak. The ROC curve provides an evaluation of the discrimination quality of a peak. In addition, the area under the curve (AUC) was determined and only peaks with AUC ≥ 0.80 values were selected. The principal component analysis (PCA) was used to evaluate the distinguishing capability between drug-resistance (or virulence) phenotype/genotype profiles in the isolates based on their peptide mass fingerprints.

The sensitivity, specificity, positive predictive value (PPV), and negative predictive value (NPV) were calculated for the potential biomarker peaks.

### Peptide/protein identity assignment

MS spectra were first processed using flexAnalysis software (Bruker Daltonics) in which top-hat baseline subtraction and spectra smoothing were performed. The spectra were then exported to the Biotools software (Bruker Daltonics), in which protein/peptide identification was performed by latter submitting the data to the Mascot Server (Matrix Science, Boston, USA) and run against the SwissProt database for peptide/protein assignment.

## Supplementary information

Supplementary Information

Dataset S3
