# Supplementary material for: Screening of biomarkers of drug resistance or virulence in ESCAPE pathogens by MALDI-TOF mass spectrometry
Source: Sci Rep. 2019 Dec 12;9:18945. doi: 10.1038/s41598-019-55430-1 (PMC6908712; doi:10.1038/s41598-019-55430-1)
Supplement: Supplementary file 1 — Supplementary Information [file 41598_2019_55430_MOESM1_ESM.docx]

**Supplementary Information**

**Article in Scientific Reports**

Screening of biomarkers of drug resistance or virulence in ESCAPE pathogens by MALDI-TOF mass spectrometry.

*Samantha Flores-Treviño, Elvira Garza-González, Soraya Mendoza-Olazarán, Rayo Morfín-Otero, Adrián Camacho-Ortiz, Eduardo Rodríguez-Noriega, Adrián Martínez-Meléndez, and Paola Bocanegra-Ibarias.*

**Supplementary Figure S1.** MS spectrum sample of each species.


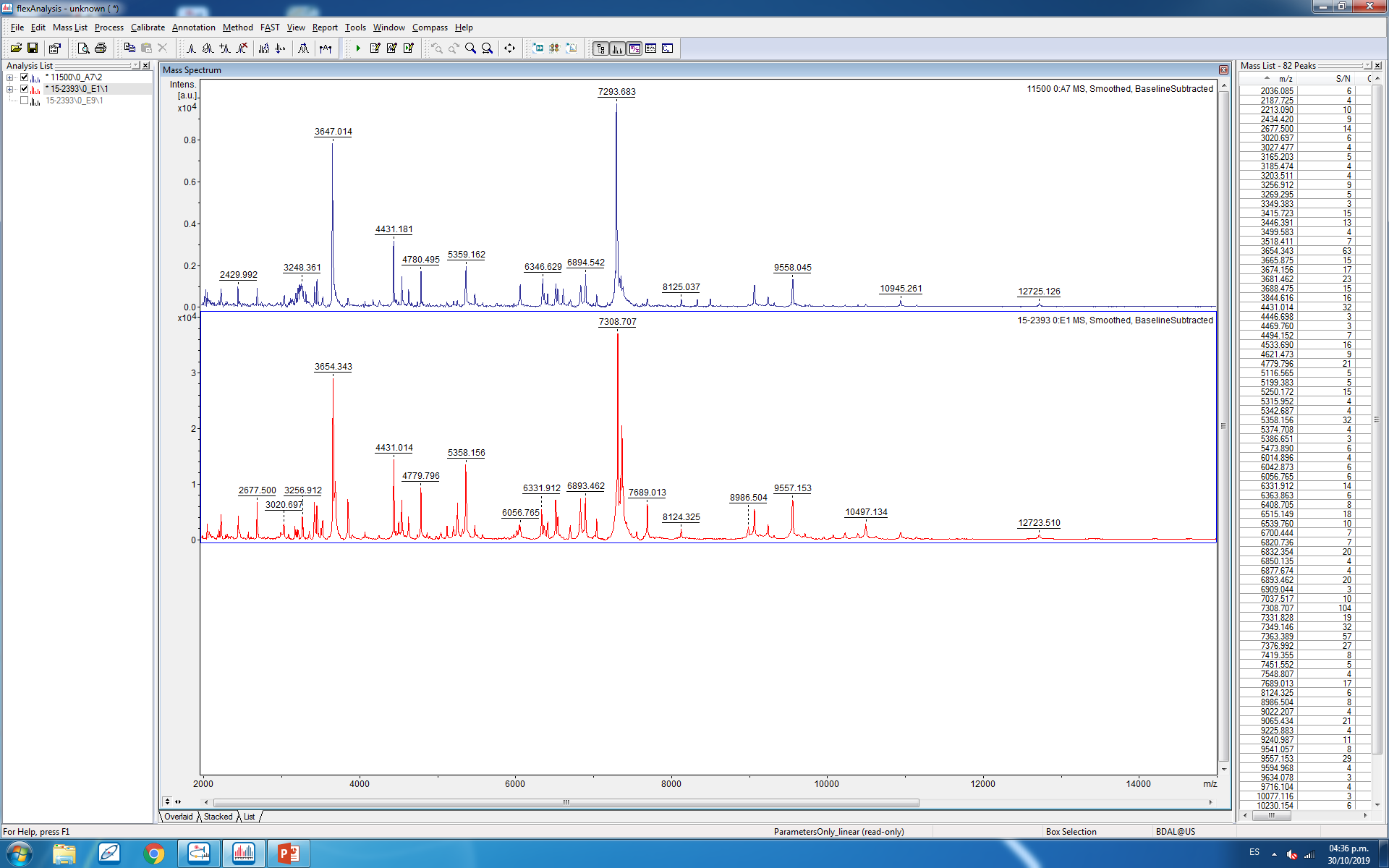

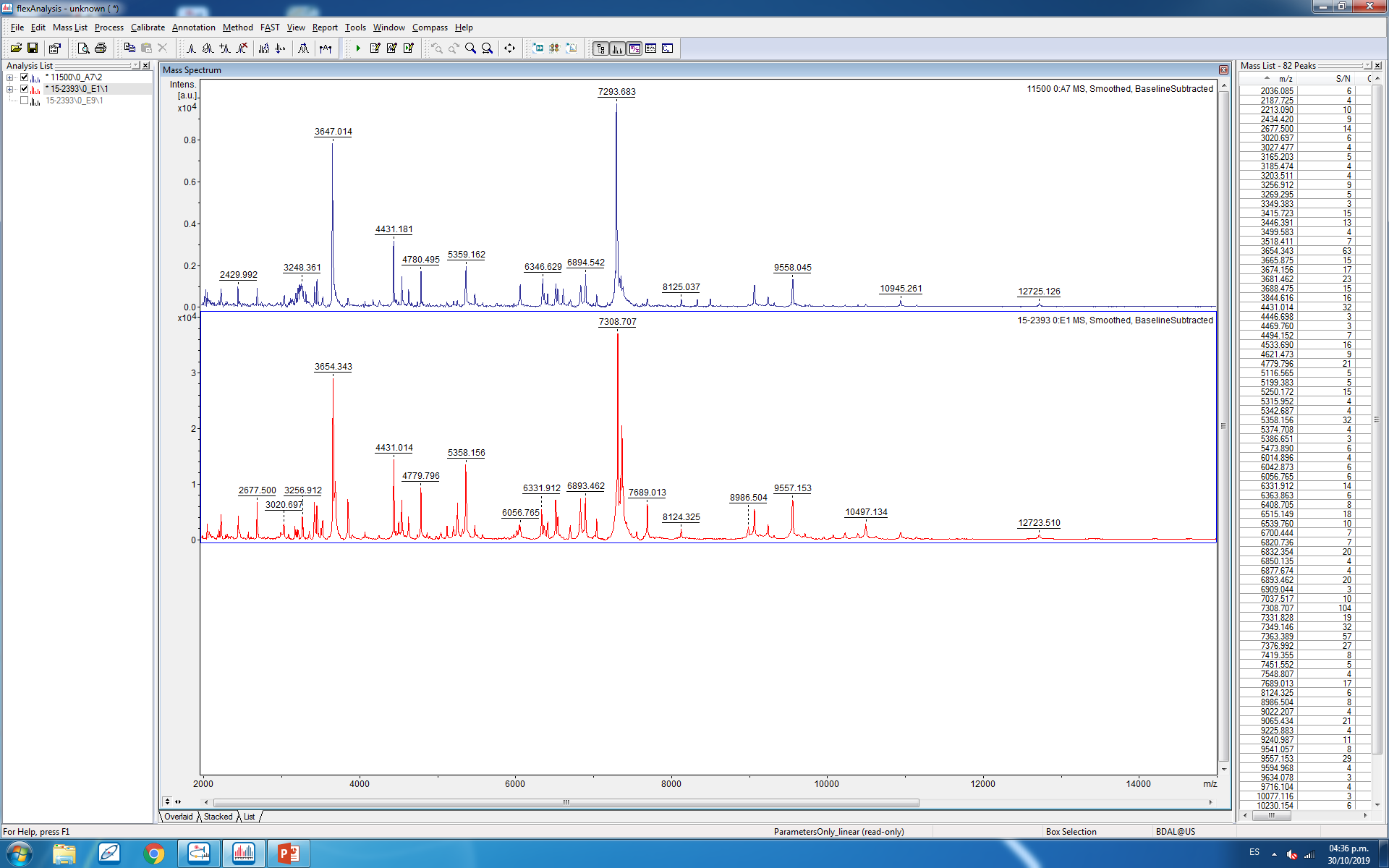


A

B

C


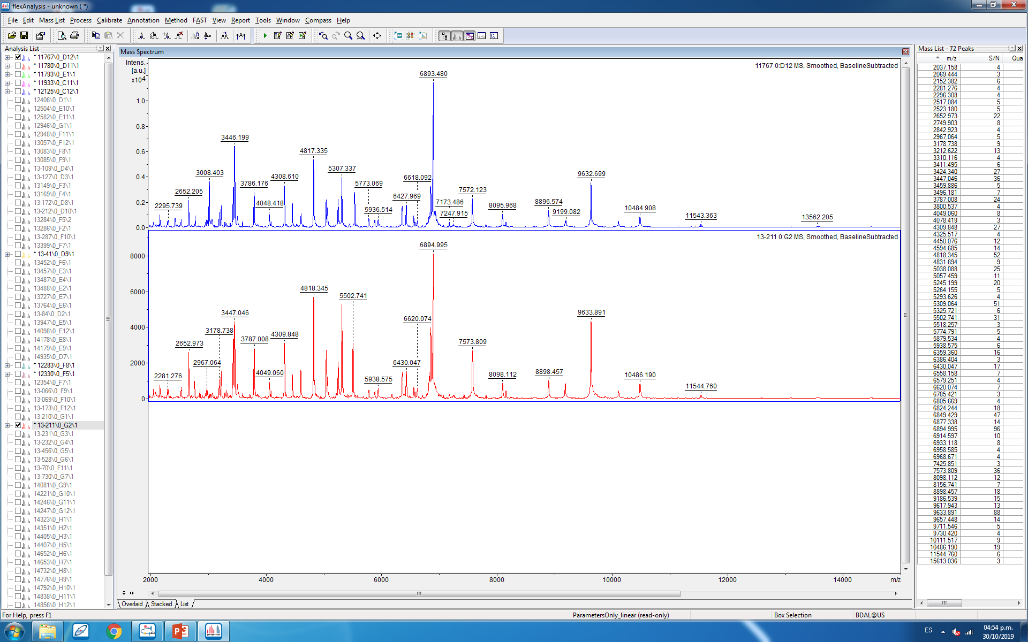

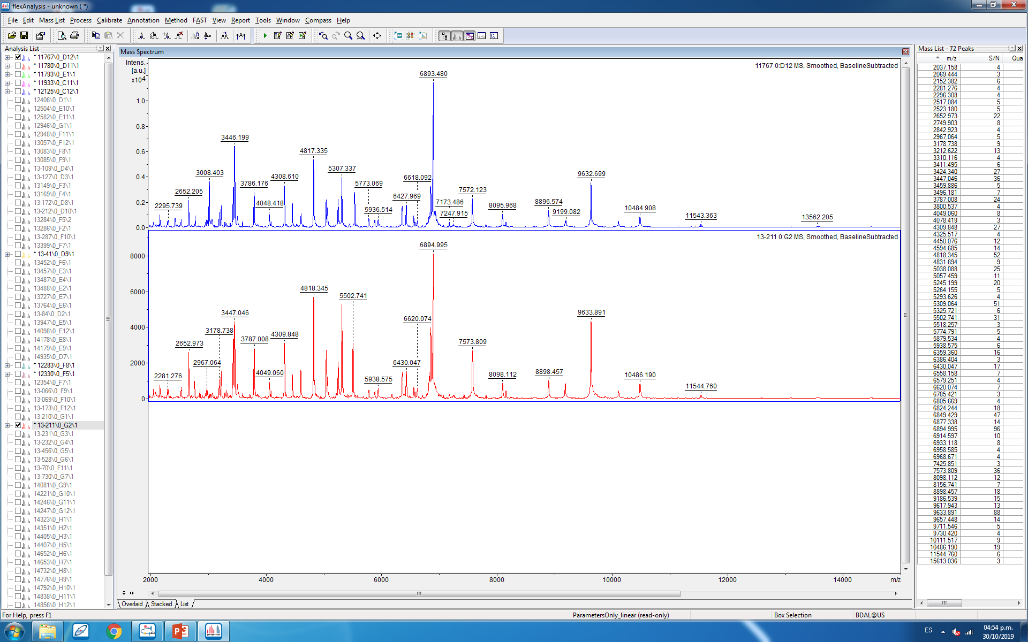

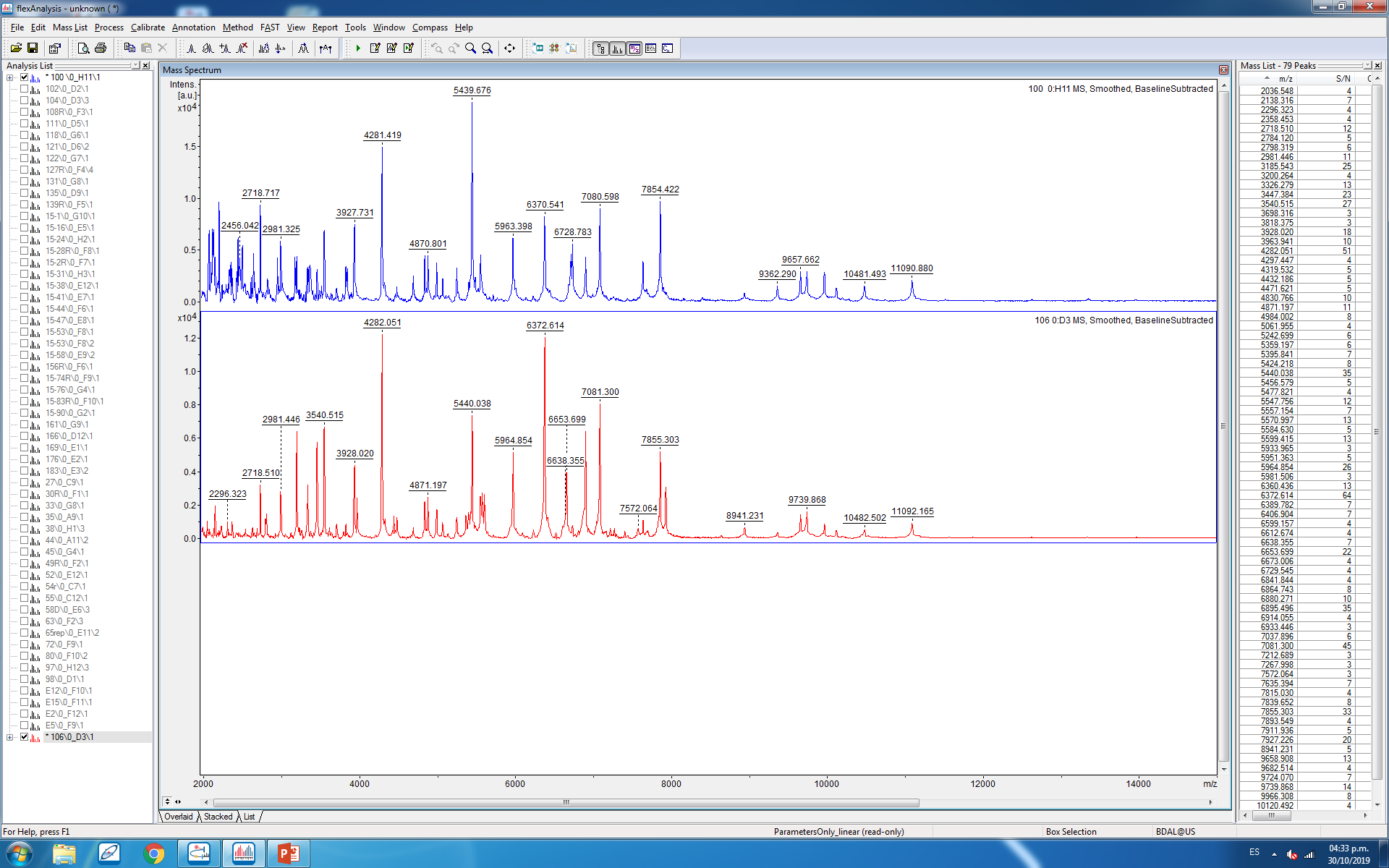

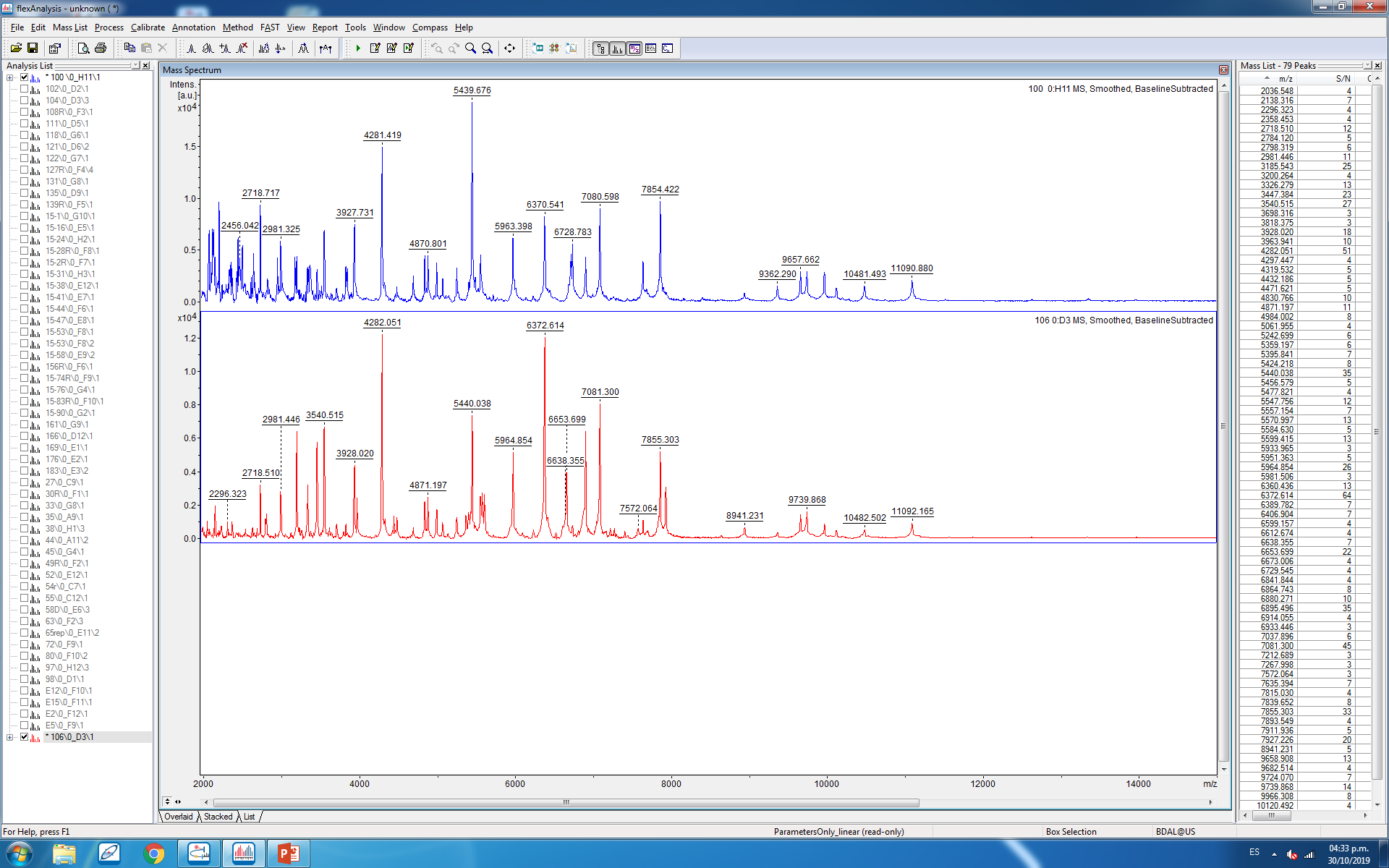


D

E

F


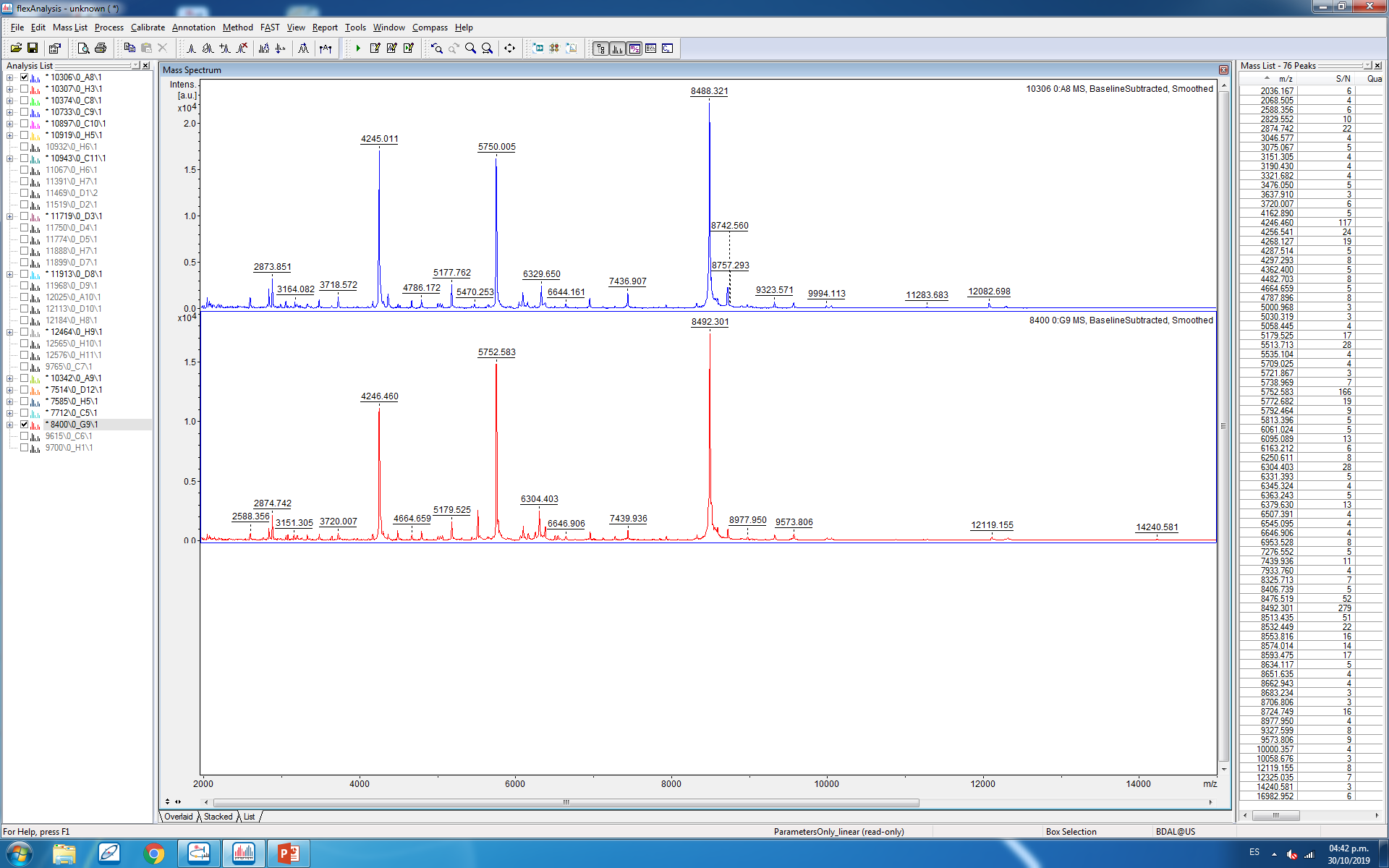

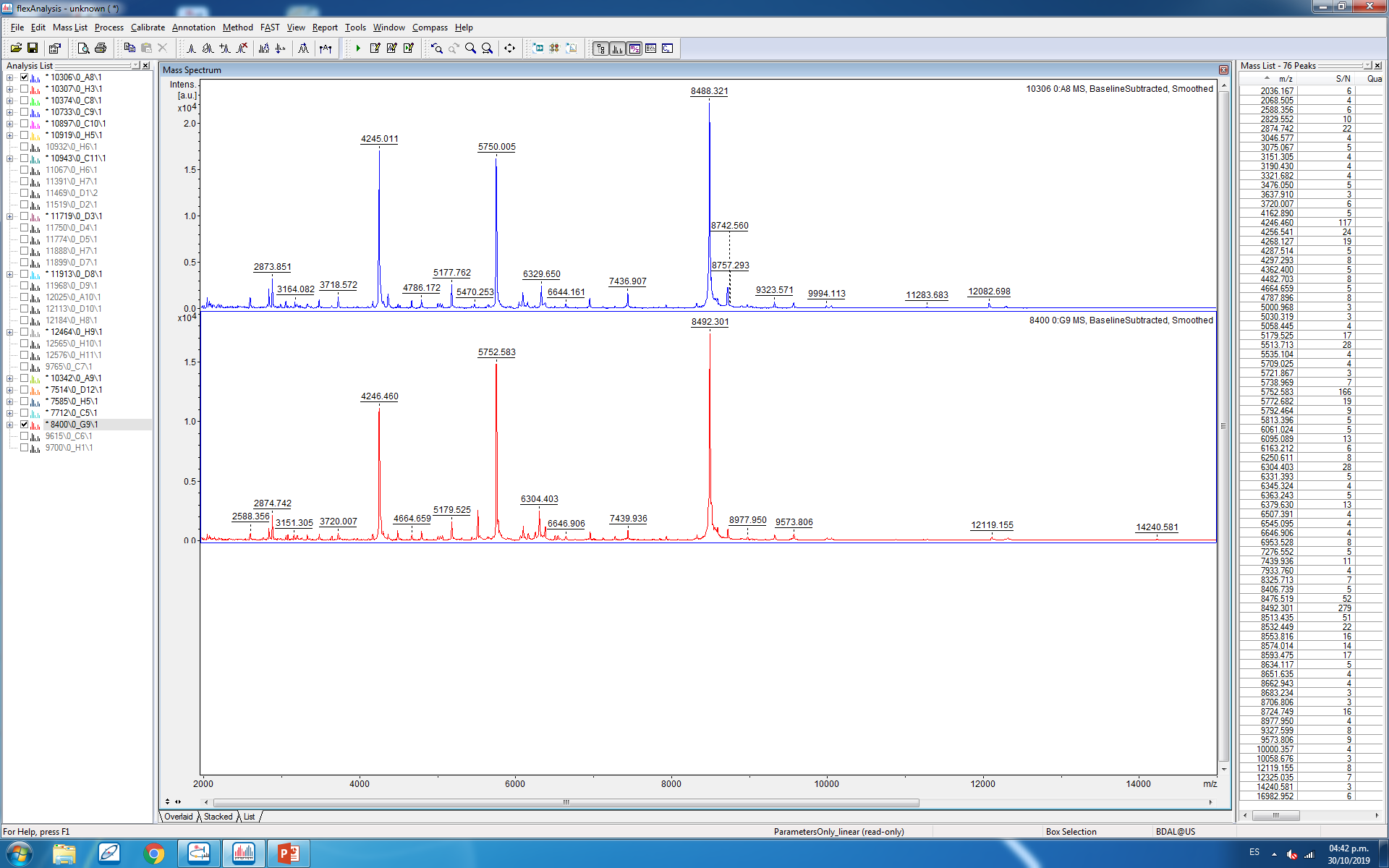

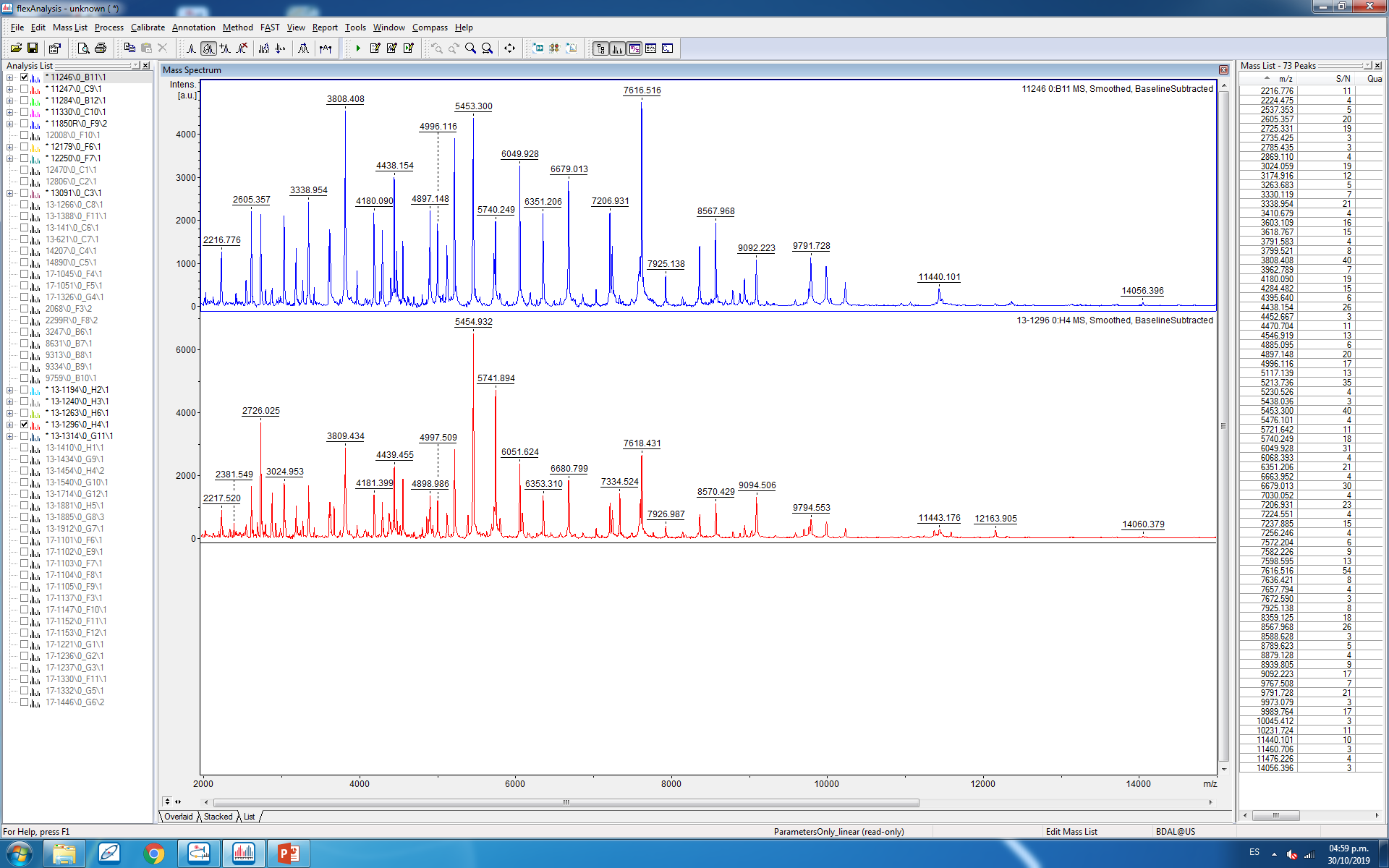

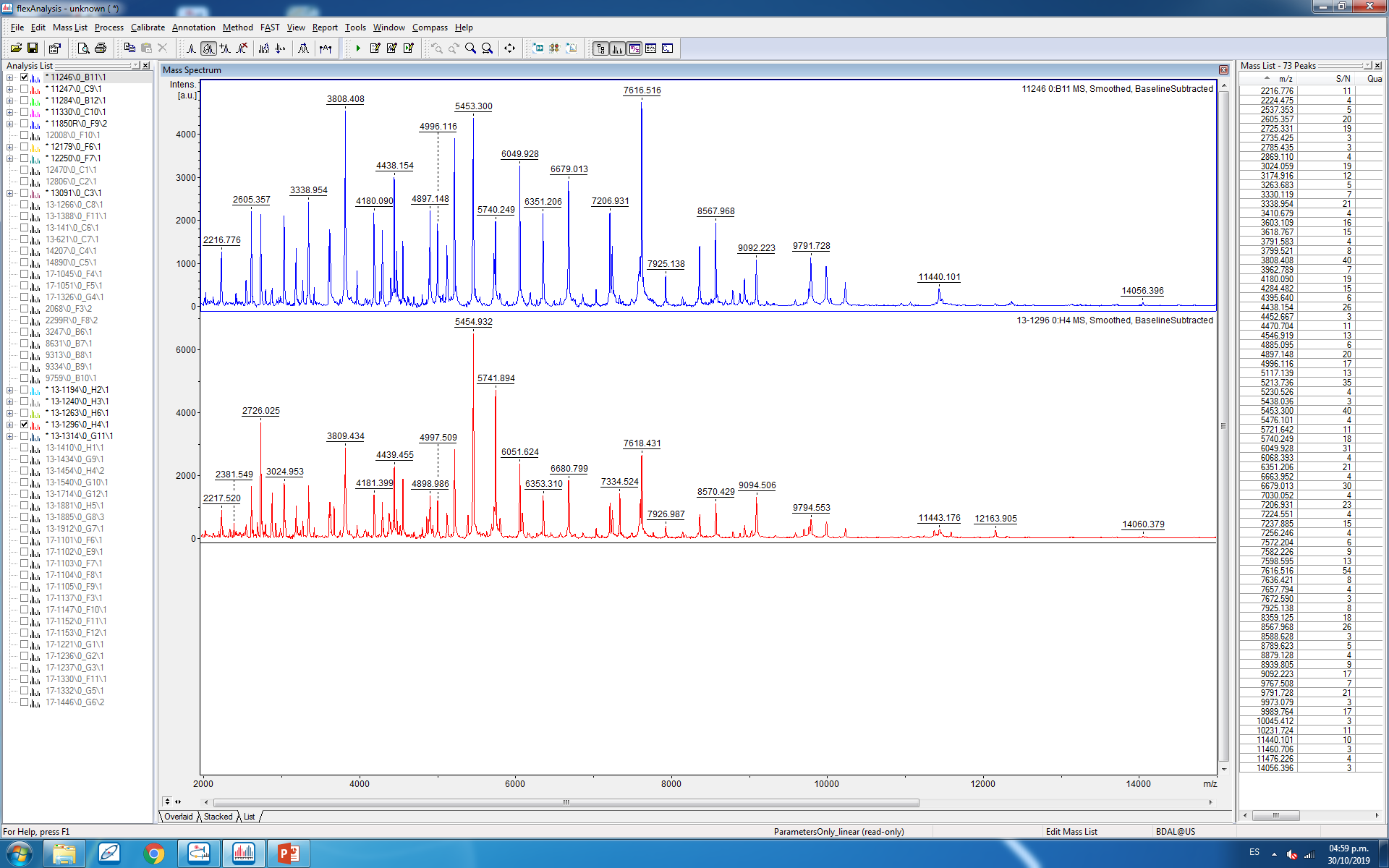

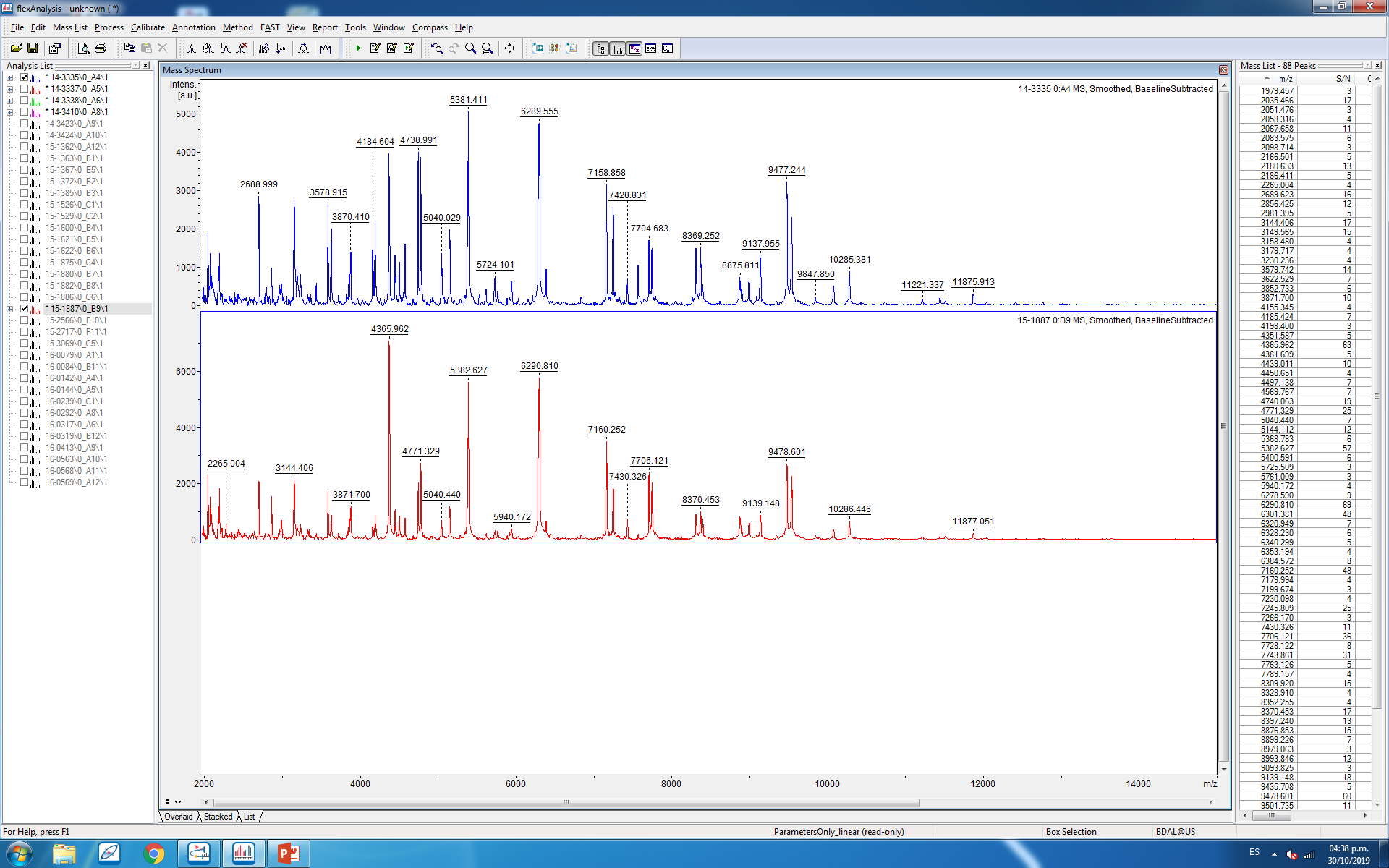

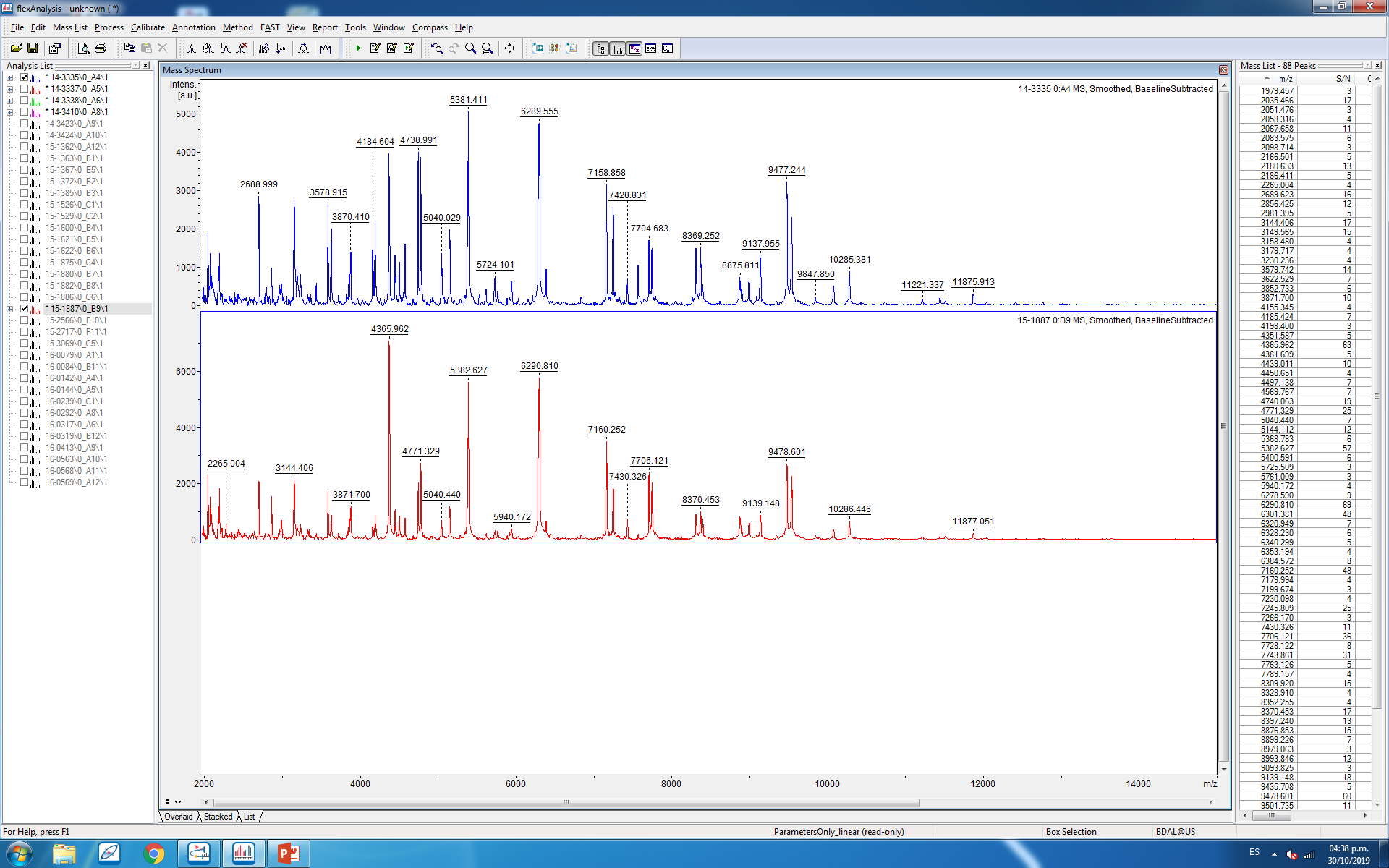


MS spectrum sample of each ESCAPE species (a) *E. faecium* resistant (blue) and susceptible (red) to vancomycin; (b) *S. aureus* resistant (blue) and susceptible (red) to methicillin; (c) *C. difficile* ribotype 027 (blue) and non-027 (red); (d) *A. baumannii bla*_OXA-24/58_ positive (blue) and *bla*_OXA-24/58_ negative (red); (e) *P. aeruginosa* MDR (blue) and non-MDR (red); (g) *K. pneumoniae* resistant (blue) and susceptible (red) to carbapenems.

**Supplementary Table S2.** Performance of three model algorithm to classify spectra.

GA: genetic algorithm; QC: quick classifier; MDR: multidrug resistant; SNN: supervised neural network.

| **Species** | **Algorithm** | **Cross-validation (%)** | **Recognition capability (%)** | **Correct classified part of valid spectra (%)** | |
| --- | --- | --- | --- | --- | --- |
| *E. faecium* |  |  |  | **Vancomycin-resistant** | **Vancomycin-susceptible** |
|  | GA | 90.8 | 100.0 | 100.0 | 100.0 |
|  | SNN | 93.9 | 98.4 | 100.0 | 96.8 |
|  | QC | 96.9 | 98.5 | 97.1 | 100.0 |
| *S. aureus* |  |  |  | ***mecA* positive** | ***mecA* negative** |
|  | GA | 95.3 | 98.6 | 97.2 | 100.0 |
|  | SNN | 87.7 | 100.0 | 100.0 | 100.0 |
|  | QC | 87.1 | 97.2 | 94.4 | 100.0 |
|  |  |  |  |  |  |
| *C. difficile* |  |  |  | **027** | **Non-027** |
|  | GA | 90.1 | 97.2 | 100.0 | 94.4 |
|  | SNN | 90.7 | 97.8 | 98.3 | 97.2 |
|  | QC | 93.6 | 96.6 | 94.8 | 94.4 |
|  |  |  |  |  |  |
| *A. baumannii* |  |  |  | ***bla*_OXA-24_ positive** | ***bla*_OXA-58_ positive** |
|  | GA | 71.0 | 92.9 | 100.0 | 85.7 |
|  | SNN | 59.7 | 51.9 | 3.8 | 100.0 |
|  | QC | 94.6 | 94.2 | 88.5 | 100.0 |
|  |  |  |  | ***bla*_OXA-24/58_ positive** | ***bla*_OXA-24/58_ negative** |
|  | GA | 50.6 | 82.5 | 78.8 | 86.1 |
|  | SNN | 68.2 | 82.5 | 78.8 | 86.1 |
|  | QC | 50.9 | 72.0 | 60.6 | 83.3 |
| *P. aeruginosa* |  |  |  | **MDR** | **Non-MDR** |
|  | GA | 76.6 | 100.0 | 100.0 | 100.0 |
|  | SNN | 84.6 | 98.2 | 100.0 | 96.4 |
|  | QC | 78.6 | 89.2 | 92.6 | 85.7 |
| *K. pneumoniae* |  |  |  | **Carbapenem-resistant** | **Carbapenem-susceptible** |
|  | GA | 78.1 | 99.0 | 98.0 | 100.0 |
|  | SNN | 83.9 | 95.0 | 100.0 | 90.0 |
|  | QC | 82.8 | 90.4 | 85.7 | 95.0 |
|  |  |  |  | **Carbapenem-/colistin-resistant** | **Carbapenem-/colistin-susceptible** |
|  | GA | 86.0 | 94.4 | 93.8 | 95.0 |
|  | SNN | 67.1 | 88.8 | 87.5 | 90.0 |
|  | QC | 74.6 | 83.1 | 81.3 | 85.0 |

**Supplementary Dataset S3.** Statistically significant peaks (*p* ≤ 0.05) detected for carbapenem-resistant *K. pneumoniae* (n=96), *C. difficile* ribotype 027 (n=76), carbapenem and colistin-resistant *K. pneumoniae* (n=75), VREfm (n=70), MDR *P. aeruginosa* (n=61), MRSA (n=45), *bla*_OXA-58_ *A. baumannii* (n=6), and carbapenem-resistant *A. baumannii* (*bla*_OXA-24_ or *bla*_OXA-58_; n=2).

**Supplementary Figure S4.**

A

C

B

G

F

E

D

H

Principal Component Analysis (PCA) of ESCAPE species. The analysis of subgroups of specific phenotypes in all ESCAPE species is included for (a) *E. faecium* resistant (red) and susceptible (green) to vancomycin; (b) *S. aureus* resistant (red) and susceptible (green) to methicillin; (c) *C. difficile* ribotype 027 (red) and non-027 (green); (d) *A. baumannii bla*_OXA-58_ (green) and *bla*_OXA-24_ (red); (e) *A. baumannii bla*_OXA-24/58_ positive (green) and *bla*_OXA-24/58_ negative (red); (f) *P. aeruginosa* MDR (red) and non-MDR (green); (g) *K. pneumoniae* resistant (red) and susceptible (green) to carbapenems; (h) *K. pneumoniae* resistant (red) and susceptible (green) to carbapenems/colistin.
